# Successful Use of Extracorporeal Membrane Oxygenation for Respiratory Failure Caused by Mediastinal Precursor T Lymphoblastic Lymphoma

**DOI:** 10.1155/2014/804917

**Published:** 2014-12-17

**Authors:** Masafumi Oto, Kyoko Inadomi, Toshiyuki Chosa, Shima Uneda, Soichi Uekihara, Minoru Yoshida

**Affiliations:** ^1^Department of Hematology and Medical Oncology, Japanese Red Cross Kumamoto Hospital, 2-1-1 Nagamine-minami, Higashi-Ku, Kumamoto 861-8520, Japan; ^2^Department of General Internal Medicine, Japanese Red Cross Kumamoto Hospital, 2-1-1 Nagamine-minami, Higashi-Ku, Kumamoto 861-8520, Japan

## Abstract

Precursor T lymphoblastic lymphoma (T-LBL) often manifests as a mediastinal mass sometimes compressing vital structures like vessels or large airways. This case was a 40-year-old male who developed T-LBL presenting as respiratory failure caused by mediastinal T-LBL. He presented with persistent life threatening hypoxia despite tracheal intubation. We successfully managed this respiratory failure using venovenous (VV) ECMO. Induction chemotherapy was started after stabilizing oxygenation and the mediastinal lesion shrank rapidly. Respiratory failure caused by compression of the central airway by tumor is an oncologic emergency. VV ECMO may be an effective way to manage this type of respiratory failure as a bridge to chemotherapy.

## 1. Introduction

Precursor lymphoid neoplasm is a highly aggressive neoplasm comprised of immature B or T cells. Precursor T lymphoblastic lymphoma (T-LBL) patients usually present with systemic lymphadenopathy. About 50 to 75 percent of patients with precursor T-LBL have mediastinal lesions [1]. Most patients with mediastinal lesions have a bulky disease and sometimes develop complications such as tracheal obstruction and superior vena cava syndrome. Hypoxia caused by massive atelectasis is often refractory to administration of oxygen due to a ventilation-perfusion (V/Q) mismatch leading to complete shunt of the lung. The use of ECMO in life threatening hypoxia due to malignancy is rare. We report a case of a 40-year-old male with T-LBL presenting as severe hypoxia successfully managed by ECMO.

## 2. Case Report

A 40-year-old male was admitted to hospital because of chest discomfort and dyspnea. The patient had been well until 2 weeks before admission, when chest pain, cough, and dyspnea had developed. Two days before admission, he could not lie down because of dyspnea. He went to another hospital and a chest X-ray (CXR) was performed. A CXR revealed a widened mediastinum and he was referred to the emergency department of our hospital.

**Figure 1:**
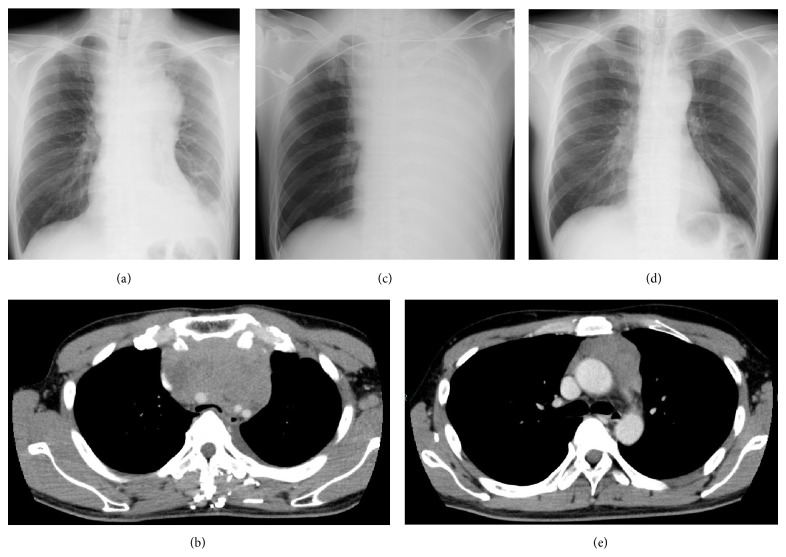
A CXR performed on admission shows a widened mediastinum (a). A contrast enhanced CT on admission shows a mediastinal tumor compressing trachea (b). A CXR performed after intubation on day 2 shows atelectasis of the left lung and a venous cannula inserted for ECMO (c). A CXR performed after one course of consolidation chemotherapy on day 71 shows disappearance of atelectasis and shrinkage of the mediastinal tumor (d). A contrast enhanced CT on hospital day 87 shows partial remission of the mediastinal tumor (e).

On examination, he appeared dyspneic and presented with orthopnea. His vital signs were normal. He had no peripheral lymphadenopathy. Laboratory tests on admission showed elevated lactate dehydrogenase level of 744 IU/L. The result of arterial blood gas was normal (Table 1). A CXR revealed a widened mediastinum and left pleural effusion (Figure 1(a)) and contrast enhanced computed tomography (CT) of the chest showed a mediastinal mass of 12 cm by 7 cm, compressing the trachea (Figure 1(b)). A CT revealed no lymphadenopathy other than the mediastinal lesion.

On hospital day 2, tracheal intubation was performed by an anesthesiologist as the patient was considered to have high risk of suffocation. During the tracheal intubation, the left main bronchus was obstructed completely by a mediastinal tumor and the patient suddenly developed hypoxia. Oxygen was administered through a tracheal tube but the oxygen saturation was approximately 80 percent. ECMO was introduced immediately to recover oxygenation, placing a venous cannula in the right common femoral vein and an arterial cannula in the jugular vein. A CXR after intubation revealed atelectasis of the left lung (Figure 1(c)). After introducing ECMO, oxygen saturation was maintained at about 98 percent and PaO_2_ was 96 mmHg (Table 1). Percutaneous fine needle biopsy of the mediastinal tumor was performed and the patient was transferred to the intensive care unit. A provisional pathological diagnosis was LBL. On hospital day 3, chemotherapy, comprised of adriamycin, vincristine, cyclophosphamide, and prednisolone (CHOP), was started to debulk the mediastinal lesion.

After chemotherapy was started, the mediastinal mass shrank rapidly and oxygenation gradually improved. He was weaned from ECMO on hospital day 8 and weaned from the mechanical ventilation and was extubated on hospital day 28. He did not have any respiratory sequelae after extubation. The final pathological diagnosis was T-LBL. Chromosomal analysis did not reveal any abnormalities and an FISH of the BCR-ABL was negative. Acute Lymphoblastic Leukemia 202 protocol without imatinib (Japan Adult Leukemia Study Group) [2] was started on hospital day 23 followed by subsequent consolidation chemotherapy. A CXR performed on day 71 shows disappearance of atelectasis and shrinkage of the mediastinal tumor (Figure 1(d)). A contrast enhanced CT of the chest performed on hospital day 87 confirmed partial remission of the tumor (Figure 1(e)). The patient's sister was confirmed as having full-matching HLA and the patient was referred to another hospital to receive allogeneic hematopoietic transplantation.

Adverse reactions during clinical courses were febrile neutropenia, acute kidney injury, which did not require renal replacement therapy, and right femoral subcutaneous abscess caused by cannulation for ECMO. He did not develop bleeding or thromboembolism as complications of ECMO.

## 3. Discussion

Obstruction of the airway is an oncologic emergency that requires immediate intervention. Malignant lymphoma and non-small-cell lung cancer are the major causes of superior vena cava (SVC) syndrome [3] and these cancers also cause obstruction of the central airway. Acute onset massive atelectasis sometimes causes hypoxia, which is resistant to the administration of oxygen. The mechanism of refractory hypoxia is a physiologic shunt of the lung with maintained blood flow, but no lung ventilation. Obstruction of the trachea by a tumor is usually managed with high dose steroids and concurrent radiation therapy or stenting of the trachea. However, life threatening severe hypoxia, as in this case, cannot await the effect of these therapies and more aggressive respiratory support is mandatory. This is a rare case report of mediastinal T-LBL presenting with respiratory failure, in which chemotherapy was administered using ECMO.

ECMO is a mechanical cardiopulmonary support, which is usually applied intraoperatively to facilitate cardiac surgery. There are two types of ECMO, venoarterial (VA) and venovenous (VV). The VA ECMO provides hemodynamic plus respiratory support and the VV ECMO provides respiratory support only. Recent data showed favorable outcome by using ECMO in adult patients with severe acute respiratory failure [4] and in patients with respiratory failure caused by H1N1 influenza infection [5]. ECMO has been used increasingly in patients with ARDS since these positive results were reported. Recently, some reports have been published on the use of ECMO in respiratory failure associated with hematological malignancy. Wohlfarth et al. reported 14 cases of respiratory failure managed by ECMO in hematological malignancy. Two patients with obstruction of the airway received induction chemotherapy using ECMO with successful results [6]. Furthermore, other case reports showed that respiratory failure such as ARDS or idiopathic pneumonia could be managed with ECMO [7–9]. ECMO dramatically improved oxygenation in this case and enabled the patient to receive chemotherapy, and he was weaned from ECMO after shrinkage of the tumor. We believe that ECMO should not be applied to respiratory failure of advanced solid tumors as non-small-cell lung cancer because these tumors have limited sensitivity to chemotherapy.

A major complication from ECMO is bleeding and thromboembolism [10]. Major bleeding events were documented in 36 percent of cases when ECMO was used in patients with hematological malignancy, which is associated with a high mortality rate [6]. We have to pay special attention to bleeding when we use ECMO in patients with a hematological malignancy because these patients have hemorrhagic diathesis as a symptom of the underlying disease.

In conclusion, we report a case of mediastinal T-LBL presenting as severe hypoxia successfully managed by ECMO. ECMO may be an effective way to manage respiratory failure as a bridge to chemotherapy in carefully selected patients with hematological malignancies.

## Figures and Tables

**Table 1:** Results of arterial blood gases and oximetry.

Variable	On admission	2nd day (before induction of ECMO)	2nd day (after induction of ECMO)
Arterial blood gases and oximetry			
Fraction of inspired oxygen	0.21	1.0	1.0
Base excess (mmol/liter)	0.8		−1.2
pH	7.41		7.50
Partial pressure of carbon dioxide (mm Hg)	40		27
Partial pressure of oxygen (mm Hg)	70		96
Bicarbonate (mmol/liter)	25.4		24.2
Oxygen saturation (%)	94	80	98
